# Septicaemia with deep venous thrombosis and necrotising pneumonia caused by acute community-acquired methicillin-resistant *Staphylococcus aureus* in an infant with a three-year follow-up: a case report

**DOI:** 10.1186/s12879-022-07166-z

**Published:** 2022-02-24

**Authors:** Mei Lu, Meijiao Fu, Yanhong Zhang, Tong Shen, Hui Xie, Dengli Liu

**Affiliations:** 1 Department of Paediatrics, Women and Children’s Hospital, School of Medicine, Xiamen University, Xiamen, 361003 Fujian China; 2 Department of Ultrasound, Women and Children’s Hospital, School of Medicine, Xiamen University, Xiamen, 361003 China; 3 Department of Paediatrics, The First Affiliated Hospital of Xiamen University, Xiamen, 361003 Fujian China

**Keywords:** Community-acquired methicillin-resistant staphylococcus aureus, Deep venous thrombosis, Necrotising pneumonia, Collateral circulation, Case report

## Abstract

**Background:**

Community-acquired methicillin-resistant *Staphylococcus aureus* (CA-MRSA) is a common pathogen that usually causes bacteraemia, osteomyelitis, as well as skin and soft tissue infections. However, deep venous thrombosis (DVT) and necrotising pneumonia are rare in infants.

**Case presentation:**

We report the case of a one-month-five-day-old girl who was hospitalised for DVT and necrotising pneumonia due to septicaemia associated with *Staphylococcus aureus*. She recovered after treatment with intravenous antibiotics and multiple anticoagulant therapy, but DVT persisted at the three-year follow-up. Collateral circulation around the DVT was well-formed. Post thrombotic syndrome was not observed.

**Conclusions:**

*Staphylococcus aureus* complicated by DVT and necrotising pneumonia is rare and can be successfully treated.

## Background

Deep venous thrombosis (DVT) is rare in infants. The incidence of venous thromboembolism (VTE) in children aged 1 month to 18 years was estimated to be 0.07 per 10,000 and 0.14 per 10,000 children in Canada and the Netherlands, respectively [1, 2]. VTE in newborns or children is associated with infection, heart disease, immobility, surgery, and central venous catheterisation [2]. Septic thrombophlebitis is often associated with bacteraemia caused by adjacent infections, such as community-acquired methicillin-resistant *Staphylococcus aureus* (CA-MRSA) [3]. For example, Lemierre’s syndrome is defined as thrombophlebitis of the internal jugular vein following pharyngitis [4]. *Staphylococcus aureus* (*S. aureus*) is a known cause of Lemierre’s syndrome, which results in necrotising pneumonia and septic internal jugular vein thrombosis [5]. *S. aureus* is a common pathogen that causes bacteraemia, osteomyelitis, and skin and soft tissue infections [6]. CA-MRSA was recognised worldwide in the late 1990s as a cause of life-threatening disseminated infections with high morbidity and mortality related to Panton-Valentine leucocidin (PVL) virulence and the staphylococcal cassette chromosome mec type IV [7, 8]. The incidence of CA-MRSA pneumonia is very low, with a conservative estimated prevalence of 0.51–0.64 cases per 100,000 [9]. Necrotising pneumonia, characterised by the destruction of the parenchymal structure and the formation of a cavity in liquefied necrosis, is a rare and serious condition for which the main causative pathogens are *Streptococcus pneumoniae*, *S. aureus*, and *Mycoplasma pneumoniae* [10].

Here, we describe a previously healthy infant with DVT and necrotising pneumonia caused by severe CA-MRSA infection, including detailed clinical information, manifestations, treatment, and the three-year follow-up.

## Case presentation

A one-month-five-day-old previously healthy female patient presented to the emergency department with a chief complaint of fever for five hours in October 2017. Her temperature peaked at 39.2℃. She had no symptoms of cough, diarrhoea, or rash. Her complete blood count revealed a white blood cell (WBC) count of 7.90 × 10^9^/L (normal, 4.5 ~ 12 × 10^9^/L) and elevated levels of C-reactive protein (CRP) at 63.40 mg/L (normal, 0 ~ 8 mg/L). She was admitted and underwent a detailed physical examination and laboratory tests. Empirical cefotaxime sodium administration was initiated. Four hours after admission, she presented with irritability and tremors, with a temperature of 39.0 °C, heart rate of 200 beats/min, systolic blood pressure of 86 mmHg, diastolic blood pressure of 52 mmHg, respiratory rate of 50 breaths/min, and oxygen saturation of 95% on room air. Chest X-ray (CXR) showed no abnormal signals. The right lower limb was found to become swollen, and cyanosis occurred simultaneously. The skin temperature and tension of the affected right limb increased significantly compared to that of the left. The circumferences of the right thigh and left thigh were 22 cm and 16 cm, respectively. No visible lesions appeared anywhere on her skin.

### DVT and treatment

**Fig. 1:**
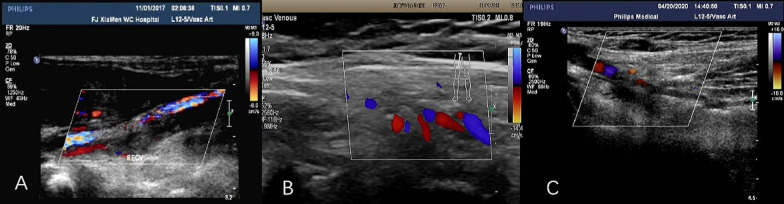
Ultrasound imaging of DVT. **A** Thrombus (Day 1) visualised in the right common femoral vein and superficial femoral vein of the right thigh. **B** (2 months later) Collateral circulation was formed. **C** (2 years 5 months later) Thrombus persisted in the right common femoral vein and superficial femoral vein

Coagulation analyses reported a D-dimer level of 4257 ng/ml (normal, 0 ~ 500 ng/ml), a prothrombin time (PT) of 11.9 s (normal, 9.4 ~ 12.5 s), International Normalized Ratio (INR) of 1.10 (normal, 0.88 ~ 1.16), a partial thromboplastin time (APTT) of 27.1 s (normal, 25.1 ~ 36.5 s), and fibrinogen (Fib) of 4.39 g/L (normal, 2 ~ 4 g/L). Based on these findings, 25 IU/kg of unfractionated heparin (UFH) was delivered intravenously to prevent disseminated intravascular coagulation (Table 1). CXR and ultrasonography revealed subcutaneous soft tissue swelling with subcutaneous cellulitis and no signs of osteomyelitis. Colour Doppler ultrasonography revealed a hypoechoic mass from the right external iliac vein to the proximal superficial femoral vein of the right thigh without signals of blood flow, and the maximal diameter of the femoral vein lumen had expanded to 0.22 cm, which confirmed a diagnosis of DVT (Fig. 1A). No abnormal Doppler signals were detected in the left lower extremity or in other vessels. She was transferred to the paediatric intensive care unit. Intravenous injection of urokinase was administered for thrombolytic therapy, and the dose was adjusted from 2000 to 4000 IU/kg·d for a total of four days based on the infant's symptoms and changes in thrombosis. At the same time, UFH was correspondingly modified to 20 IU/kg·h for 14 days, then 10 IU/kg·h for one day and back to 20 IU/kg·d for 20 days. A multidisciplinary team discussion deemed it was impossible to perform intra-catheter thrombolysis on a one-month-old infant in that state. After four days of combined treatment with urokinase and heparin, the circumference of the right thigh reduced from 22 to 21 cm, and the bruising was improved. On day seven of hospitalisation, the patient was given aspirin (30 mg/kg ·d) for 3 days and 5 mg/kg ·d for 28 days because her symptoms overlapped with those of Kawasaki disease, which could not be ruled out. Oral warfarin was administered for three months after discharge (Table 1). Ultrasonography was performed to monitor the changes in DVT on days 3, 5, 9, 16, and months 1, 2, 6, 12, 20, and 29 after thrombosis was diagnosed. The thrombus in the right external iliac vein disappeared after five days, but persisted in the right femoral vein. The lumen diameter reduced from 0.22 to 0.20 cm by day 16, and to 0.15 cm at 1 month. Collateral circulation formed beside the right common femoral vein and superficial femoral vein two months later (Fig. 1B). Two years and five months later, the thrombus in the right femoral vein showed little change (Fig. 1C). No gangrene occurred. Neither magnetic resonance imaging nor computed tomography (CT) of the right lower limb was performed on this patient.

**Table 1:** Time and results of the coagulation function test and the corresponding anticoagulant therapy of the patient

	INR	PT (s)	APTT (s)	Fib (g/L)	D-dimer (ng/ml)	Platelet^a^ (10^9^ /L)	UFH	Urokinase (IU/kg d)	Aspirin (mg/kg d)	Warfarin (mg/d)
Day 0^b^	1.10	11.9	27.1	4.39	4257	447	25 IU/kg	–	–	–
Day 1	1.31	14.1	47.9	5.06	3750	217	20 IU/kg/h	2000	–	–
Day 2	1.14	12.3	34.9	6.15	3586	272	20 IU/kg/h	3000	–	–
Day 3	0.99	10.7	31.6	6.39	2921	318	20 unit/kg/h	4000	–	–
Day 4	1.06	11.4	33.7	5.51	2841	413	20 unit/kg/h	4000	–	–
Day 7	1.36	14.6	42.3	3.6	2600	786	20 unit/kg/h	–	30	–
Day 10	1.17	12.6	40	3.16	5375	457	20 unit/kg/h	–	5	–
Day 14	1.03	11.1	28	3.16	3086	871	10 unit/kg/h	–	5	
Day 15	–	–	–	–	–	792	20 unit/kg/d	–	5	
Day 35	0.89	9.6	31.3	2.89	2728	610	Stop	–	5	0.5 mg/d
4 months	1.13	12.2	38.3	1.42	46	425	–	–	–	Stop

^a^Normal range, 150 ~ 450*10^9^ /L; ^b^day of admission

### Antimicrobial therapy and pulmonary complications

**Fig. 2:**
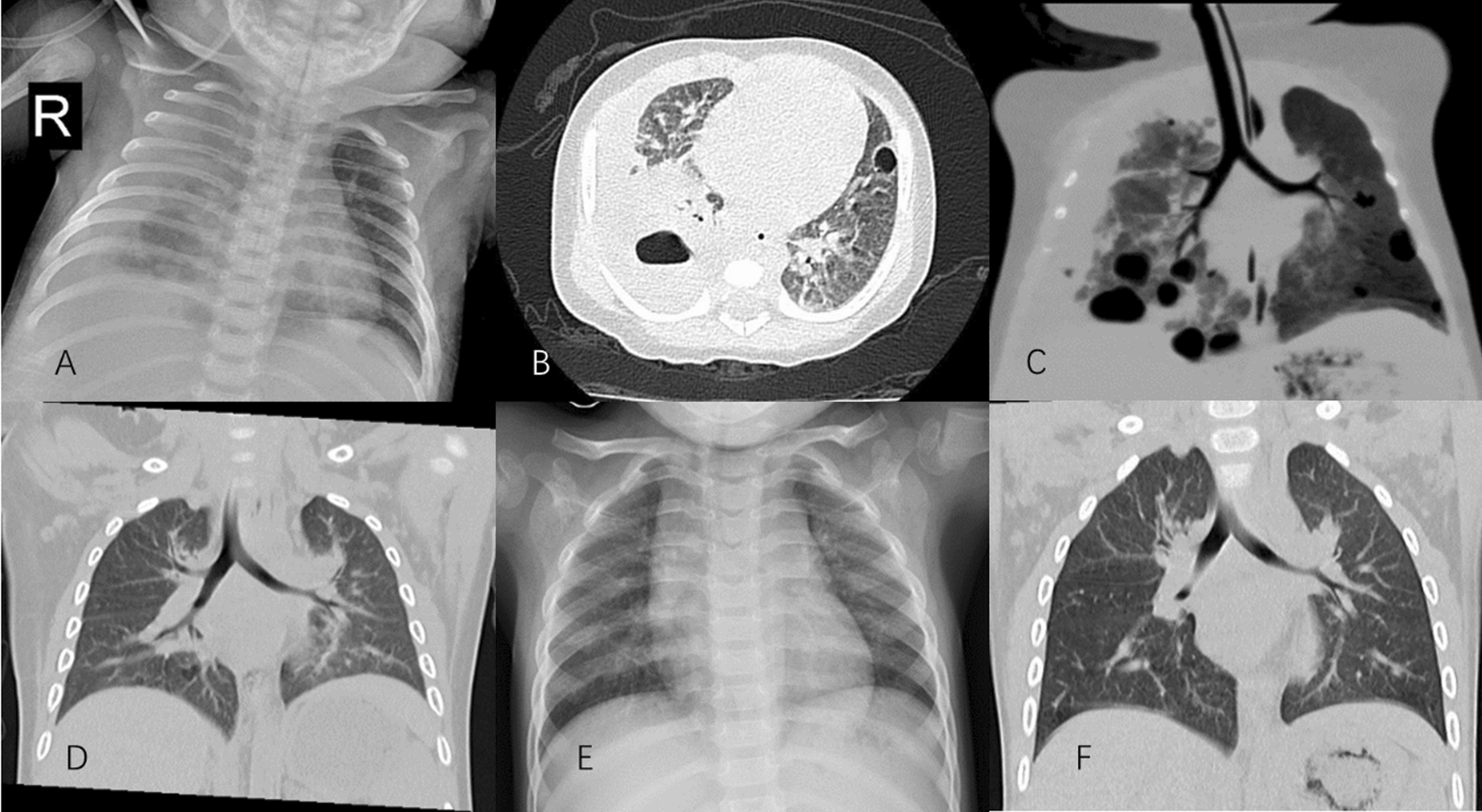
Changes on CXR and lung CT. **A**–**C** (Day7) CXR showing right pleural effusion and suspected lung abscess. CT revealing multiple pulmonary loculated abscesses, cavitation, consolidation, and right pleural effusions. **D** (CT 1 year later), **E** (CXR 2 years later) and **F** (CT three years later) showed significant improvement

Cefotaxime was used empirically and changed to vancomycin (60 mg/kg d) and meropenem (45 mg/kg d) for severe unexplained infection 4 h after admission. Two days after admission, two sets of blood cultures from different sites identified methicillin-resistant *Staphylococcus aureus* (MRSA), which was sensitive to vancomycin, and the meropenem was subsequently discontinued. The vancomycin trough concentration was 14.2 μg/ml (normal, 10–20 μg/ml). Her symptoms improved after antibiotic therapy, but her temperature reached 38.5 °C once or twice a day. On day seven of her hospital stay, her cough was obvious, breathing became fast, and she had decreased breath sounds on the right side of her lungs. Her oxygen saturation decreased to 90% on room air and was 98% on mask oxygen inhalation. CXR revealed multiple suspected lung abscesses and right pleural effusions (Fig. 2A). A lung CT scan revealed right pleural effusions and multiple abscesses in both lungs, especially on the right side, indicating necrotising pneumonia (Fig. 2B, C). Thoracentesis and closed chest drainage lasted for five days. After a total of 60 ml of purulent exudate was drained, and her temperature and breath returned to normal after one day of drainage. Her pleural fluid was yellow and had 21,008 leukocytes per ml (89.4% neutrophils), lactate dehydrogenase of 2755 U/L, total protein of 47.2 g/L (albumin 20.9 g/L, globulin 26.3 g/L), and glucose of 5.09 mmol/L. Fluid culture also showed MRSA sensitivity to vancomycin. Repeated lung CT scans later showed that the pulmonary lesions improved during convalescence (Fig. 2A–F). There were no signs of pulmonary embolism, and pulmonary CT angiography was not performed. Vancomycin was administered for 35 days, and renal impairment did not occur.

### Other lab findings

Initial laboratory findings on the first day of hospitalisation showed a procalcitonin of 3.07 ng/mL (normal, 0–0.25 ng/mL) and lactic acid (1.94 mmol/L (normal, 0–2.0 mmol/L). Six days after admission, the total homocysteine level was 6.7 μmol/L (normal, 0–15 μmol/L), antithrombin III was 35.6% (normal, 75–125%), protein S was 87.4% (normal, 60–130%), and protein C was 20% (normal, 70–140%). A small amount of lupus anticoagulant was present, which was considered a part of the severe sepsis. Protein C recovered to 89.2% after treatment. Acquired protein C deficiency was also established. Repeated echocardiography did not find evidence of endocarditis.

### Follow-up

The infant had septicaemia due to *S. aureus*, followed by deep vein thrombosis, necrotising pneumonia, multiple lung abscesses, and pyothorax. The patient was discharged after 35 days. She is now over 3 years old, with normal growth and development. Lung CT scans showed significant improvement, DVT still exists, and the nearby collateral circulation was good. The circumference of the right thigh was 1.0 cm larger than the left thigh. No complications of post-thrombotic syndrome or reinfections occurred, and there was no effect on the patient’s quality of life.

## Discussion

*S. aureus* is a common pathogenic bacterium in clinics. The main invasion route of *S. aureus* is through the skin, and the main complications include empyema, pleural effusion, perirenal abscess, liver abscess, pericardial effusion, endocarditis, meningitis, septic shock, arthritis, and hematopoietic dysfunction [6, 11]. *S. aureus* can produce a variety of toxins, enzymes, and antigen proteins, including membrane-damaging toxins, causing cell death through interference with receptors or secreting enzymes that damage biological membranes [12]. PVL, one of the toxins produced by *S. aureus*, increases pathogenicity by necrosis, accelerates apoptosis, and destroys polymorphonuclear and mononuclear cells [8]. These factors lead to ischaemic necrosis of the lung tissue or necrotising pneumonia. In a 5-year study in France, PVL was positive in 13 children hospitalised for *S. aureus* necrotizing pneumonia [13]. In another study, four of 75 patients were diagnosed with necrotising pneumonia [14]. *S. aureus* was found in both the blood culture and pleural effusion culture in our patient, however, PVL detection could not be performed due to the limitations of our hospital. In our study, necrotising pneumonia was not considered because of the lack of dyspnoea in the first week until she developed a cough and persistent fever. Early CXR re-examination may help identify lung complications. CT can more clearly reflect pathological changes and identify lesions more accurately than CXR. Early initiation of adequate vancomycin therapy and closed thoracic drainage are pivotal to improving the outcomes of MRSA pneumonia.

DVT is another formidable threat caused by MRSA, which is exceedingly rare and serious in the paediatric population [15]. DVT occurs mainly because of the use of central venous catheters in intensive care units, trauma, overwhelming sepsis, and underlying congenital diseases [16, 17]. In a study by Hoppe et al., only one patient had DVT simultaneously, and it was associated with extensive skin and soft tissue infections surrounding the involved vessels [14]. Our case had no evidence of osteomyelitis, local skin abscesses, central venous catheters, trauma, or congenital disease. Septicaemia of MRSA, which is considered a perianal skin infection, is the most important risk factor for DVT. PVL of *S. aureus* also plays a significant role in smooth muscle spasm and the aggregation of platelets and enzymes, which can interact with fibrinogen, resulting in plasma clotting, venous stasis, and DVT [15]. DVT is mainly characterised by limb swelling, pain, and superficial vein irritation; however, it is not typical in infants. In our case, osteomyelitis and soft tissue infection of the right lower limb were initially suspected. The purple and swollen skin, remarkable elevation of D-dimer, and severe infection caught our attention, and Doppler ultrasonography examination of the right deep vein was performed. DVT was diagnosed, and dynamic changes were monitored using Doppler ultrasonography due to its non-invasive and non-radiation characteristics.

There is no optimal treatment for venous thrombosis in paediatric patients, and the treatments for VTE in different paediatric departments vary [18]. Most recommendations for antithrombotic therapy in paediatric have only weak supporting evidence [16]. Determining the treatment presented the biggest challenge with this case. Experience and consensus on the treatment of infective thrombosis in infants are limited, and we referred to the 3rd Guidelines for the diagnosis and treatment of deep vein thrombosis in the Chinese Journal of Vascular Surgery [19]. UFH, low-molecular-weight heparin, and oral vitamin K antagonists are the most common anticoagulants, however, determining their optimal dose and duration is a challenging dilemma in children [20, 21]. The suggested duration of anticoagulation was between 6 weeks and three months when there are identified risk factors, such as septic thrombophlebitis, family history of thrombophilia, cancer, chronic inflammatory disease, immobility, trauma to the affected site, or the presence of a central line [16, 21]. In our study, the patient was healthy and not immunocompromised, and there were no acquired prothrombotic disorders such as sickle cell disease, carbohydrate-deficient glycoprotein syndrome, or nephrotic syndrome. Although several anticoagulant drugs were used, DVT did not disappear completely, and collateral circulation was formed. Surgical thrombectomy is an effective method for thrombus removal. However, the age of onset in this child was barely more than 1 month, and surgical thrombectomy was not yet possible.

D-dimer is a fibrin degradation product produced by the dissolution of the fibre protein complex in the hypercoagulable state. When DVT occurs, measuring the D-dimer level is helpful [16]. However, D-dimer levels also increase in critical conditions and malignant tumours [22]. D-dimer has high sensitivity and poor specificity, and can be used to screen for acute VTE [23]. There are few studies on the diagnostic accuracy of D-dimer levels in children. In a study of 526 children with suspected pulmonary embolism, 34 were diagnosed with a mean D-dimer level of 2104 ± 1394 ng/ml [24]. In our study, the D-dimer levels were significantly increased. After the inflammation was controlled, the D-dimer level gradually decreased to normal, although the thrombus persisted.

In conclusion, it is rare for healthy infants to simultaneously have severe CA-MRSA sepsis, DVT, and necrotising pneumonia. After the combined treatment, the prognosis of necrotising pneumonia was good. DVT does not completely disappear and requires long-term follow-up.

## Data Availability

All data generated or analysed during this study are included in the published article.
